# ASO Author Reflections: Outcome-Based Referrals for Parathyroidectomy may be the Future of Care for Patients with Primary Hyperparathyroidism

**DOI:** 10.1245/s10434-025-17797-6

**Published:** 2025-09-07

**Authors:** David Rekhtman, Rachel Kelz

**Affiliations:** Department of Surgery, Hospital of the University of Pennsylvania, Philadelphia, USA

## Past

Traditionally, surgeon volume has been used as a proxy metric to represent postsurgical outcomes in patients undergoing parathyroidectomy for primary hyperparathyroidism. Studies from the early 2000s laid the groundwork for the use of volume as a predictor of postoperative complications, allowing for subsequent studies to assess whether particular patient populations, such as those with negative imaging, would benefit from a more “experienced” surgeon.^1^ However, the cutoff for a “low-volume” versus a “high-volume” surgeon remains difficult to assess.^2^ The American Association of Endocrine Surgeons recommends parathyroidectomies to be conducted by surgeons with an annual case rate of more than ten operations per year.^3^ The challenge of utilizing outcomes has been twofold. First, as opposed to using reported volumes from a surgeon or institution, case mix varies, requiring statistical adjustment for comparative assessment. Second, it is statistically challenging to interpret complication rates for low-volume surgeons, because the lack of complications, or the presence of a single complication, may disproportionately skew their overall outcomes. In an era with increased reporting and data-sharing, referring patients to facilities with superior reported outcomes may become possible.

## Present

We developed a simulation model to measure the impact of optimizing parathyroidectomy care patterns based on serious adverse events based on facility-reported outcomes.^4^ Wilson score estimates were used to develop more accurate predicted confidence intervals for the complication rate of low-volume centers; this novel statistical technique, which, to our knowledge, has not previously been applied in clinical research, allowed for the inclusion of low-volume centers performing at least ten operations per year in our analysis.^5^ With the use of five different state datasets, we trained the model using the reported parathyroidectomy outcomes of 11,733 patients to then simulate referrals to higher-performing centers for 3005 patients. We found that simulated care resulted in lower serious adverse events and lower rates of 30-day readmissions without a meaningful difference in cost compared with the original outcomes. Although higher-volume centers correlated with lower complication rates, patient outcomes between facilities with the same volume varied greatly, highlighting the advantage of referral practices that are based on outcomes and not solely volume.

## Future

To achieve outcome-based referral practices, a few changes in both research and clinical practice will be required. A formalized, measurable definition for “resolution of hyperparathyroidism”—or “cure”—needs to be developed to allow for accurate measurement and reporting of parathyroidectomy outcomes. The current ICD-10-based system reinforces a focus on complication rates without consideration for operative success. However, postoperative complications may affect quality of life to a greater extent than the symptoms of hyperparathyroidism, so both are important to consider in future studies. Once reporting of outcomes becomes standard practice, primary care physicians and endocrinologists would be able to refer patients to surgeons based on what matters the most to them whether it be outcomes, communication style, or other nonclinical factors. We hope to have made the case for the development and use of outcome-based metrics to benefit patients undergoing parathyroidectomy for primary hyperparathyroidism.
